# Transcriptome Response of Female *Culicoides sonorensis* Biting Midges (Diptera: Ceratopogonidae) to Early Infection with Epizootic Hemorrhagic Disease Virus (EHDV-2)

**DOI:** 10.3390/v11050473

**Published:** 2019-05-24

**Authors:** Dana Nayduch, Vijay Shankar, Mary K. Mills, Tanner Robl, Barbara S. Drolet, Mark G. Ruder, Erin D. Scully, Christopher A. Saski

**Affiliations:** 1 Arthropod-borne Animal Diseases Research Unit, USDA-ARS, 1515 College Avenue, Manhattan, KS 66502, USA; barbara.drolet@usda.gov; 2 Clemson University Center for Human Genetics, 152 Self Regional Hall, 114 Gregor Mendel Circle, Greenwood, SC 29649, USA; vshanka@clemson.edu; 3 Department of Biology and Geology, University of South Carolina Aiken, 471 University Parkway, Aiken, SC 29801, USA; marymi@usca.edu; 4 School of Medicine, University of Kansas Medical Center, 3901 Rainbow Boulevard, Kansas City, KS 66160, USA; trobl@kumc.edu; 5 Southeastern Cooperative Wildlife Disease Study, Department of Population Health, College of Veterinary Medicine, University of Georgia, Athens, GA 30602, USA; mgruder@uga.edu; 6 Stored Product Insect and Engineering Research Unit, USDA-ARS, 1515 College Avenue, Manhattan, KS 66502, USA; erin.scully@usda.gov; 7 Department of Plant and Environmental Sciences, Clemson University, Clemson, SC 29634, USA

**Keywords:** *Culicoides*, vector, arbovirus, differential expression, dissemination, innate immune, visual perception, midge, epizootic hemorrhagic disease virus

## Abstract

Female *Culicoides sonorensis* biting midges are vectors of epizootic hemorrhagic disease virus (EHDV), which causes morbidity and mortality in wild and domesticated ruminants. The aims in this study were to identify key changes in female midge transcriptome profiles occurring during early infection with EHDV-2. Midges were fed either negative control bloodmeals or bloodmeals containing EHDV-2 and transcriptomes were acquired at 36 h through deep sequencing. Reads were *de novo* assembled into a transcriptome comprised of 18,754 unigenes. Overall, there were 2401 differentially expressed unigenes and ~60% were downregulated in response to the virus (953 up; 1448 down). Downstream Gene Ontology enrichment, KEGG pathway mapping, and manual analyses were used to identify the effect of virus ingestion at both the gene and pathway levels. Downregulated unigenes were predominantly assigned to pathways related to cell/tissue structure and integrity (actin cytoskeleton, adherens junction, focal adhesion, hippo signaling), calcium signaling, eye morphogenesis and axon guidance. Unigenes attributed to sensory functions (especially vision), behavior, learning and memory were largely downregulated. Upregulated unigenes included those coding for innate immune processes, olfaction and photoreceptor pigments. Our results suggest that midges respond to virus infection as soon as 36 h post-ingestion, and that EHDV-2 may have a significant phenotypic effect on sensory and neural tissues.

## 1. Introduction

Female *Culicoides sonorensis* (Wirth and Jones) are vectors of orbiviruses including epizootic hemorrhagic disease virus (EHDV) [1,2]. In the USA, EHDV causes moderate to severe disease in ruminants, and can be especially pathogenic to captive and wild cervids, such as white-tailed deer in non-endemic areas [3,4,5]. Of the seven serotypes of EHDV, EHDV-1, -2 and -6 are endemic in the USA, and EHDV-1 and -2 have been associated with cyclical disease outbreaks in ruminants for over 60 years [1]. Data from the Southeastern Cooperative Wildlife Disease Study (SCWDS) collected during a 2012 epidemic of hemorrhagic disease in wild ruminants (e.g., white-tailed deer) in the Midwestern region of the USA demonstrated that the responsible serotypes were EHDV-2 and EHDV-6 [1]. From 2006 to 2015, the serotype most commonly isolated in ruminants or samples submitted to SCWDS was EHDV-2 (*n* = 647), with a prevalence approximately an order of magnitude greater than EHDV-6 (*n* = 84) or EHDV-1 (*n* = 38) [2].

Although *C. sonorensis* midges are confirmed vectors for EHDV [6,7,8,9], vector competence can vary among individuals. Factors underlying vector competence for EHDV have not been elucidated, but likely include physical barriers (e.g., peritrophic matrix and midgut tissues) as well as immune-mediated defenses, such as innate immune responses and siRNA pathways (reviewed in [10]). After being ingested in the bloodmeal from infected hosts, the virus must traverse and survive these physical and immune barriers in an effort to reach the hemocoel for subsequent dissemination into the body’s tissues. The time course of these events, i.e., from initial midgut infection to dissemination into body tissues including the salivary glands, has been recently elucidated by our group by employing immunohistochemical techniques [9]. While full dissemination to the salivary glands was observed by 5 days after ingesting EHDV-2 using this staining technique, dissemination from the midgut that was undetectable by this method and may occur at an earlier time. Interestingly, virus staining was also observed in the ommatidia, optic ganglia and Johnston’s organ (used in visual and auditory perception). Furthermore, this staining was associated with tissue damage to the ommatidia, suggesting the possibility of tissue restructuring or altered function [9].

We previously produced the first *de novo* transcriptome for *C. sonorensis* and elucidated whole-midge responses to diet including early and late blood and sugar feeding [11]. In a subsequent study, we annotated components of the innate immune response, including several antimicrobial peptide (AMP) effectors [12]. The primary objective of the current study was to capture the global transcriptomic response to EHDV-2 infection in the midge during early virus infection (36 h post-ingestion). Because AMPs have been implicated in mosquitoes and other vectors [13,14,15,16], assessing their differential expression during early infection was one goal of the current study. We were particularly interested in the transcriptional response of innate immune genes and associated defense pathways during early stages of infection and dissemination from the gut in order to begin to understand the genetic basis of vector competence. We additionally sought to capture differences in gene expression that may provide insights towards the other cellular responses to virus infection and dissemination, especially those related to cell structure, modeling, integrity and architecture.

## 2. Materials and Methods

### 2.1. Virus Culture

EHDV-2 was originally isolated from the spleen of a white-tailed deer in Kansas in 2012 (ID no. CC12-304). Virus was isolated in calf pulmonary artery endothelial cells (American Type Culture Collection, Manassas, VA, USA) and passed twice in baby hamster kidney cells (BHK; American Type Culture Collection) before purification as previously described [9]. Virus media referenced below was prepared as previously described [17]. Titers were determined by cytopathic effect assays (CPE) and titration via an endpoint titration method [18].

### 2.2. Culicoides sonorensis Rearing and Treatment

The AK colony of *C. sonorensis* (Wirth & Jones) biting midges used in this study is maintained at the USDA-ARS Arthropod-Borne Animal Diseases Research Unit in Manhattan, KS. Female pupae were held at 26 °C, 70–80% relative humidity, with a 12:12 h light:dark photoperiod until emergence. For oral infections, two replicate cages of non-mated female midges (3–4 day post-eclosion, >100 per cage) were allowed to feed for 1 h on a mixture of 6 mL defribrinated sheep blood (Lampire, Everett, PA, USA) containing either 4 mL EHDV-2 (10^7.0^ median tissue culture infective doses (TCID_50_)/mL virus media; EHDV-fed treatment) or blood containing 4 mL virus-free virus media (negative controls) on a membrane feeding system as previously described [9]. After feeding, midges were immobilized using carbon dioxide gas and visually assessed for successful blood feeding. In both treatments, midges that did not feed were removed from the study. Midges (*n* = 10) from the EHDV-fed treatment group were collected immediately after feeding for virus isolation and titration [8,18].

### 2.3. Culicoides sonorensis Processing for Transcriptome Analysis

Midges were maintained on water *ad libitum* until processing. At 36 h post-ingestion, midges were immobilized with carbon dioxide gas and collected for virus isolation and titration (*n* = 10 from virus treatment group) or RNA extraction (pools of *n* = 15 for each replicate of virus treatment and control groups). Midges were homogenized in Tri-Reagent (Life Technologies, Carlsbad, CA, USA) and total RNA extraction was performed using a modification of the manufacturer’s protocol that incorporated Bromo-3-chloro-propane (Molecular Research Center, Inc., Cincinnati, OH, USA) in the extraction step and on-column DNAse treatment. RNA was quantified by nanodrop (Thermo Fisher Scientific, Waltham, MA, USA) and the RNA integrity number (RIN) and the general fragment profile were acquired with a Bioanalyzer 2100 (Agilent, Santa Clara, CA, USA) using the RNA 6000 Nano kit prior to library construction. Two biological replicates of each feeding state were performed, resulting in four libraries.

### 2.4. Transcriptome Pre-Processing, Construction and Sequencing of Libraries

Standard TruSeq mRNA Library Prep procedures (Illumina, San Diego, CA, USA) were used to enrich mRNA from the total RNA libraries, to convert to cDNA and to add sequencing adapters and barcodes for multiplexing. Pooled, multiplexed transcriptome libraries were sequenced on a HiSeq2500 (Illumina) to a depth of at least 20 million paired-end reads per sample (2×125 bp PE) with v4.0 chemistry at the Hollings Cancer Institute (Medical University of South Carolina). Raw read quality assessment was performed with FASTQC (https://www.bioinformatics.babraham.ac.uk/projects/fastqc/). Read preprocessing and trimming of adapter sequences and low-quality bases (phred33) was performed with Trimmomatic software [19].

### 2.5. De novo Assembly and Annotation

High quality processed reads were first normalized to a 100× representation by exploiting abundances of unique k-mers using the pre-processing scripts that are part of the Trinity software package [20]. Normalized reads were *de novo* assembled using the default Trinity pipeline as previously described [11]. The initial build of the transcriptome assembly was filtered for transcripts with internal stop codons and non-genuine coding sequences with the TransDecoder v5.3.0 software (https://github.com/TransDecoder/TransDecoder/releases). The resulting assembly was clustered with the cdHIT program at 98% identity threshold to collapse transcripts derived from allelic variants [21]. The final transcriptome assembly was annotated using default parameters of the Trinotate pipeline (https://trinotate.github.io/), which uses evidence from the Pfam HMM database [22], SwissProt, UniProt [23], and the non-redundant protein database. Homology-based functional annotation was performed by mapping the final unigene set to the Gene Ontology resource (http://geneontology.org), Kyoto Encyclopedia of Genes and Genomes (https://www.genome.jp/kegg/; KEGG), and Pfam [22]. The annotated transcriptome was aligned to the previously assembled *Culicoides* reference transcriptome using a reciprocal BLAST approach and redundant unigenes were removed.

### 2.6. Analysis of Differential Unigene Expression and Gene Ontology Enrichment

Processed transcriptomic sequences comprised of two biological replicates per condition were aligned to the reference midge transcriptome using the GMAP short read aligner using default parameters [24]. Read counts per million (CPM) were determined with the FeatureCounts software tool [25] and output into tabular format. Differential unigene expression profiles of midge transcriptomes between EHDV-fed and negative control groups were determined using a generalized linear model (GLM) and the edgeR software package, in a pairwise manner [26]. Unigenes with |Log_2_ fold change (FC)| ≥ 1 and False Discovery Rate (FDR) corrected *p*-values of ≤0.05 were considered differentially-expressed [27]. Gene Ontology (GO) enrichment analysis was performed using GOseq [28]. Briefly, GO assignments for each unigene were extracted from the Trinotate annotation using the extract_GO_assignments_from_Trinotate_xls.pl script that is part of an associated pipeline, linking Trinotate to GOseq. Transcript lengths were determined using the fasta_seq_length.pl script from the Trinity package [20]. Differentially-expressed unigenes were first filtered for entries that contain GO terms and differentially expressed unigenes were separated into upregulated and downregulated unigene subsets. GO enrichment using GOseq was run separately on the up- and down-regulated differentially-expressed unigenes with the full list of genes with GO terms designated as the background.

### 2.7. KEGG Pathway Analysis

Specific GO terms from the enrichment analyses were selected on the strength of the enrichment (enrichment ratio and FDR-adjusted *p* value) and biological relevance to viral infection and dissemination. Unigenes associated with these enriched GO terms were further annotated using the KEGG databases. Unigenes with KEGG IDs were mapped onto KEGG pathway templates using the KEGG Mapper (www.genome.jp/kegg/mapper.html). GO terms that shared the same pathways were combined for visualization and ease of interpretation. Pathways with the largest numbers of assigned unigenes were selected for custom visualization in Adobe Illustrator (Adobe, San Jose, CA, USA). Unigenes that were part of enriched GO terms were designated as a seed within each KEGG pathway and KEGG genes within 2 degrees of connectivity from each seed were included in the mapping process resulting in a single cohesive network for each pathway instead of several small broken sections. Mapped unigenes were color coded to depict Log_2_FC in expression.

### 2.8. Analysis of Sensory, Memory and Behavior Genes

GO and Uniprot functional annotations were queried for terms related to sensory function, and unigenes that were identified as sensory-related were sub-grouped to the following categories: vision (e.g., eye development, eye morphogenesis, photoreceptor, ommatidia, etc.), olfaction (e.g., smell perception, odorant binding proteins, antenna, etc.), other sensory functions (e.g., gustatory, mechanoperception, nociception, sound perception, etc.) and brain/behavior/memory (e.g., short and long term memory, brain/CNS development and differentiation, locomotion, circadian rhythm, learning, etc.).

### 2.9. qRT-PCR Validation of Expression of Selected Unigenes

In a separate experiment, female midges (*n* = 15 per treatment, two replicates) were fed either EHDV-2 or virus-free media in blood previously mentioned, and RNA was extracted using the modified Trizol method described above. cDNA was synthesized from 500 ng total RNA using the QuantiTect Reverse Transcription kit following the manufacturer’s instructions (Qiagen, Valencia, CA, USA). qRT-PCR detection was performed using the Quanta PerfeCta SYBR green Fast Mix (Quantabio, Beverly, MA, USA), according to the manufacturer’s protocol and run in 10 µL reactions consisting of primers (Supplementary Table S1) diluted to a final concentration of 300 nM and cDNA templates diluted 1:10. To minimize variability, pipetting was performed using an Eppendorf epMotion 5070 platform and reactions run in triplicate on a Roche Lightcycler^®^ 480 with the following parameters: 95 °C for 2 min, followed by 40 cycles of 95 °C for 15 s, 55 °C for 20 s, 68 °C for 15 s and melt curve analysis. The reference gene *EF1b* [GenBank:GAWM01010754] was used, and C_T_ values were analyzed using the Relative Expression Software Tool [29], which allows for group-wise comparison and statistical analysis of relative expression while accounting for differences in primer efficiencies.

## 3. Results

### 3.1. Virus Titer

All midges fed EHDV-2 were positive for virus at 0 h post-ingestion (hpi) by CPE assay, with infectious virus titers ranging from 10^2.97^ to 10^3.3^ TCID_50_/midge at this time point. While 100% of midges collected at 36 h post-ingestion were also positive for EHDV-2 by CPE assay, 60% of midges were below the limit of detection (10^2.3^ TCID_50_/midge), with quantifiable titers between 10^2.3^ and 10^2.8^ TCID_50_/midge (Table 1).

### 3.2. Sequencing and Updating of the Culicoides sonorensis Reference Transcriptome

Deep transcriptome sequencing of *C. sonorensis* transcriptomes in this study produced a total of 153.6 million paired-end reads and 78 Gb of high-quality trimmed data. The assembled transcriptome consisted of a total of 42,326 transcripts with the sizes ranging from 224 bp to 11.8 kbp, and with an average length of 937 bp. Filtering of transcripts for valid open reading frames and a detailed manual review of homology/paralogy among our previously published unigene set [11] resulted in a final total of 18,754 unigenes. The reduction in number of recovered unigenes relative to our previous dataset is a result of deeper sequencing and more stringent filtering for redundant genes. There were 1098 unigenes unique to EHDV infection recovered in this new assembly that were not present in the previous set, and these unigenes are prepended with a “v.” (Supplementary Table S2). The updated assembly has been deposited to DDBJ/EMBL/GenBank under the accession ID GAWM00000000.2 and Bioproject PRJNA238338. The raw sequence reads have been deposited to Short Read Archives database under the accession ID SAMN11785150.

### 3.3. Unigenes That Were Differentially Expressed during Early EHDV Infection

**Figure 1:**
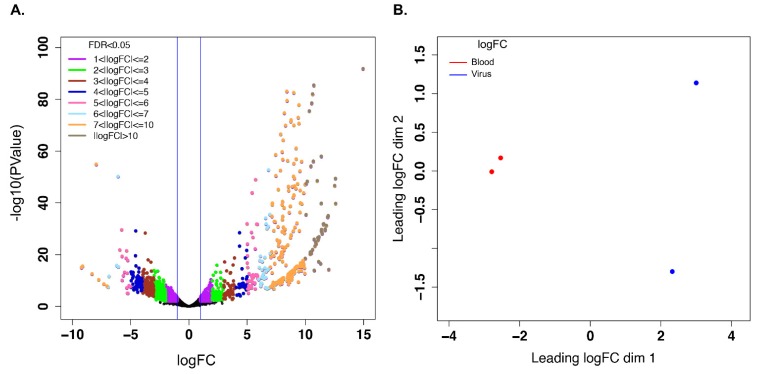
Visualization of differential gene expression analysis using edgeR. (**A**) Represents the volcano graph derived by plotting the log of the FDR-adjusted *p* value as a function of Log_2_ of the fold change (“logFC”) for each transcript in EHDV-fed midges vs. controls. (**B**) Represents the multidimensional scaling plot of the relationship matrix which is derived from Log_2_ fold change of the differentially-expressed unigenes across the sample groups showing separation of controls vs. EHDV-fed groups.

A large number of unigenes were differentially expressed in response to early infection with EHDV-2 (36 h post-ingestion) (Figure 1A). These differences are mirrored in the ordination of sample points in the multidimensional scaling (MDS) plot generated from the relationship matrix based on the Log_2_FCs of high differential expression across the two conditions (Figure 1B), and indicate separation of EHDV-fed and control groups along the dim 1 axis. Filtering the differentially-expressed unigene lists for those whose |Log_2_FC| ≥ 1 and FDR adjusted *p* ≤ 0.05 resulted in a total of 2401 unigenes. Of these, 953 unigenes were upregulated (Supplementary Table S3) and 1448 unigenes were downregulated (Supplementary Table S4) with EHDV-2 infection. A subset of both up- and down-regulated unigenes from this dataset were validated by qRT-PCR (Supplementary Figure S1). The difference in the number of unigenes between the upregulated and downregulated sets implies that infection with EHDV-2 resulted in a dominant downregulation trend (60.3%; Figure 1A). Of the upregulated unigenes, most (*n* = 387) were between 1 and 2 Log_2_FC. The remaining unigenes fell into the following categories (Log_2_FC): 2- to 4-fold, *n* = 178; 4- to 6-fold, *n* = 91; 6- to 8-fold, *n* = 129; 8- to 10-fold, *n* = 121; ≥ 10-fold, *n* = 47. Of the downregulated unigenes, the majority were downregulated between −1 and −4 Log_2_FC (total 1558 of 1782 unigenes, with 774 down −1 to −2-fold and 811 down −2 to −4-fold). The remaining downregulated unigenes fell into the following categories (Log_2_FC): −4 to −6-fold, *n* = 178; −6 to −8-fold, *n* = 38; −8 to −10-fold, *n* = 6; ≥ −10-fold, *n* = 2. Further stratification of the 2401 unigenes based on statistical significance (*p*-value brackets) resulted in 626 unigenes with *p* values between 0.05 and 0.01 (243 upregulated and 383 downregulated), 663 unigenes with *p* values between 0.01 and 0.001 (228 upregulated and 435 downregulated), 365 unigenes with *p* values between 0.001 and 0.0001 (101 upregulated and 264 downregulated) and 747 unigenes with *p* values less than 0.0001 (381 upregulated and 366 downregulated) (Supplementary Tables S2 and S3).

Of the 953 unigenes that were upregulated Log_2_FC ≥ 1, 431 had no hit to SWISS-PROT using BLASTX, 460 had no hit to SWISS-PROT using BLASTP, and 411 had no match to proteins from this database using either BLASTX or BLASTX searches. Additionally, when the Genbank nr database was queried, 259 had no match via BLASTX, 287 had no match via BLASTP, and 250 had no match to nr using either BLASTX or BLASTP. Overall, 250/953 (26%) had no match in either the SWISSPROT or nr databases, and therefore could not be functionally annotated based on homology searches. Of note, 195 of these 250 non-annotated unigenes (78%) were highly differentially-expressed, having a Log_2_FC ≥4 and merit further investigation.

**Figure 2:**
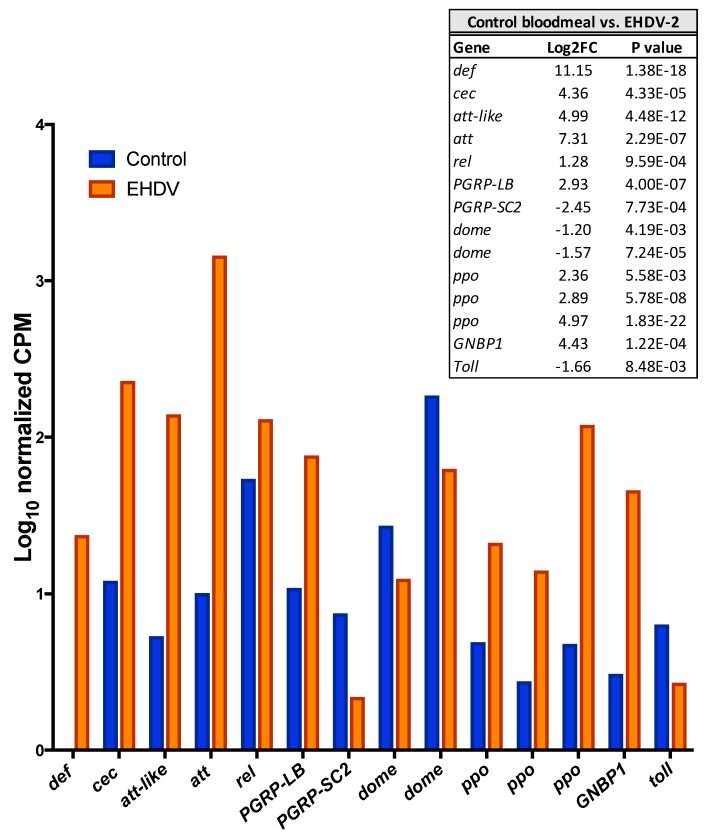
Innate immune genes that were differentially-expressed after EHDV-2 infection in the midge. Control midges were fed blood with sterile virus media and EHDV-2 midges were fed blood with virus in media (*n* = 15 per replicate, 2 replicates). Data were captured at 36 h post-ingestion for whole midges. 10-log CPM (Counts Per Million mapped reads) represents relative expression across conditions (normalized for two replicates). All unigenes shown were differentially expressed between the two conditions (*p* < 0.05). Log_2_FC and actual *p* values are shown in inset table with further details between the two conditions available in Table S2. *def, defensin* (m.9997); *cec, cecropin* (m.10000); *att-like, attacin-like* (m.3410), *att, attacin* (m.7821), *rel, relish* (m.58438), *PGRP-LB, peptidoglycan recognition protein LB* (v.751); *PGRP-SC2, peptidoglycan recognition protein SC2* (m.9236); *dome, dome* cytokine receptor (paralogs: m.63662; m.64065); *ppo, prophenoloxidase* (paralogs: m.21464, m.5965, m.41748); *GNBP1, Beta-1,3-glucan-binding protein-1* (m.20067); *toll, toll* receptor (m.9915).

Some of the upregulated unigenes that could be annotated were of particular interest with respect to the biology of the midge. Unigenes coding for several components of innate insect innate immunity including four antimicrobial peptides, the transcription factor relish, the feedback regulator PGRP-LB, three prophenoloxidase genes and GNBP1 all were significantly upregulated with EHDV-2 infection (Figure 2). Several unigenes with the greatest upregulation are involved with cell structure, especially that of axons: three isoforms of *neurobeachin* (Mean Log_2_FC for isoforms = 11), which may be involved in vesicle trafficking, membrane dynamics, cytoskeleton assembly, synaptic architecture and associative learning [30]; *longitudinals lacking* (*lola*; Log_2_FC = 12), which is a transcription factor involved in axon growth and guidance in the peripheral nervous system [31]; *dystonin* (aka *shot*; Log_2_FC = 9), which is a linker protein that integrates intermediate filaments, actin and microtubule networks that comprise the cytoskeleton, and may be integral in axonal transport [32].

Of the 1448 differentially-expressed unigenes that were downregulated Log_2_FC ≤–1, 321 had no hit in SWISS-PROT BLASTX, 311 had no hit in SWISS-PROT BLASTP, and 299 had no hit to SWISS-PROT database. In addition, 29 had no hit in nr BLASTX, 46 had no hit in nr BLASTP, and 27 had no match to proteins from this database using either BLASTX or BLASTX searches. Overall, most of the downregulated unigenes had a functional annotation, with only 27/1448 (1.9%) having no hit in either the SWISS-PROT or nr databases. While most insect innate immune components were upregulated with virus infection, some were downregulated including two copies of the JAK/STAT pathway receptor *dome*, the negative regulator of the Imd pathway, *PGRP-SC2,* and the receptor *toll* (Figure 2).

### 3.4. Gene Ontology Term Enrichment

GO terms enrichment analysis of differentially-expressed unigenes revealed a significantly greater degree of enrichment in the downregulated set compared to the upregulated set (Supplementary Tables S5 and S6). Specifically, there were 520 terms that were enriched (FDR-adjusted *p* ≤ 0.05) in the downregulated unigene set, compared to only 59 in the upregulated set. This stark difference in enrichment implies that a greater proportion of the downregulated unigenes were assigned to similar functional categories compared to the upregulated set. Many of the enriched GO terms in the downregulated unigene set belong to broad, high-level GO terms assigned to processes and components that are parts of key cellular functions such as metabolism, morphogenesis, biosynthesis, transcription and reproduction (Supplementary Tables S5 and S6). Notable GO terms enriched in the downregulated unigene set included regulation of transcription from the RNA polymerase II promoter, regulation of nitrogen compound metabolic process, post-embryonic animal morphogenesis, regulation of gene expression, regulation of transcription, imaginal disc-derived leg morphogenesis, flight, cell migration and compound-eye morphogenesis (Supplementary Table S7A). In contrast, enriched GO terms in the upregulated unigene set tended to belong to more specific biological processes such as amino-acid metabolism, proteolysis and digestion (Supplementary Table S7B). In addition to these biological processes, many molecular functions related to peptidase activity (serine-type peptidases, exo- and endopeptidases) and pheromones were also enriched in the upregulated set (Supplementary Table S7B).

### 3.5. KEGG Pathway Mapping of Unigenes Associated with Biologically-Relevant GO Terms

**Figure 3:**
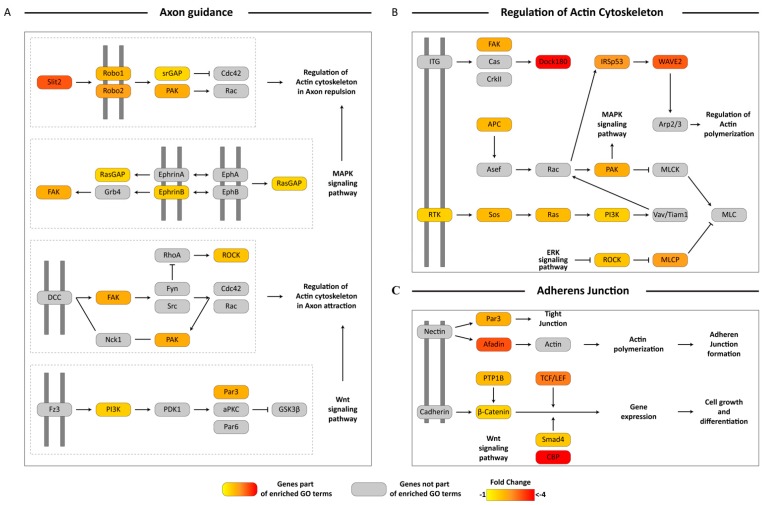
Pathway visualization using downregulated differentially-expressed unigenes in Axon guidance, Regulation of actin cytoskeleton and Adherens junction. Transcripts from enriched Gene Ontology (GO) terms from the downregulated unigene set were mapped on to KEGG Axon guidance pathway (**A**), Regulation of actin cytoskeleton (**B**) and Adherens junction (**C**) pathways and visualized manually. Boxes in color represent protein coding genes that were present within the enriched GO terms. The gradient of the color represents their fold change. Grey boxes represent protein coding genes not present in the enriched GO terms. Dotted lines represent individual modules within the parent pathway. Pathway components present on double grey lines represent cell membrane receptors. Log_2_FC differences between the two conditions can be referenced in Table S2.

**Figure 4:**
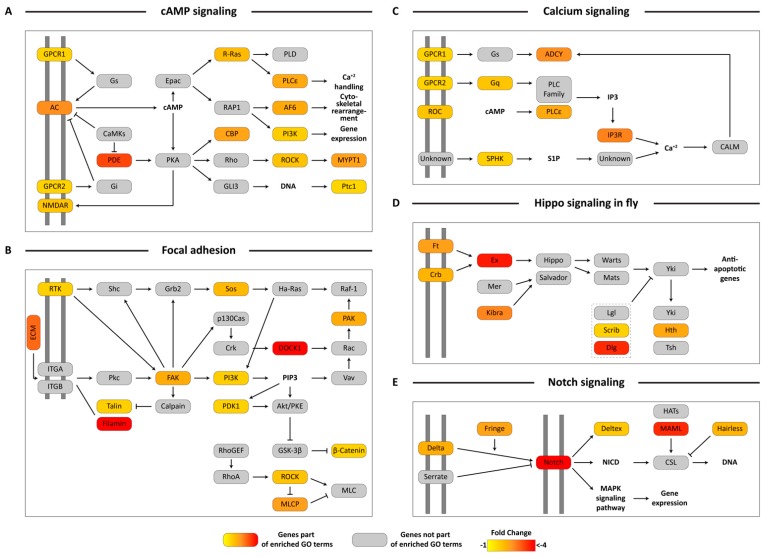
Pathway visualization using downregulated differentially-expressed unigenes in cAMP signaling, Focal adhesion, Calcium signaling, Hippo signaling in fly and Notch signaling. Transcripts from enriched Gene Ontology (GO) terms from the downregulated unigene set were mapped on to KEGG cAMP signaling (**A**), Focal adhesion (**B**), Calcium signaling (**C**), Hippo signaling in fly (**D**) and Notch signaling (**E**) pathways and visualized manually. Boxes in color represent protein coding genes that were present within the enriched GO terms. The gradient of the color represents their fold change. Grey boxes represent protein coding genes not present in the enriched GO terms. Pathway components present on double grey lines represent cell membrane receptors. Log_2_FC differences between the two conditions can be referenced in Table S2.

Analysis using KEGG pathways provided additional insight into the potential impacts of EHDV acquisition on global transcriptional profiles. Several up- or down-regulated unigenes were assigned to the same KEGG pathway thereby increasing support for potential pathway-level impacts on midges that had been exposed to EHDV. Specifically, we found several downregulated unigenes within the Axon Guidance GO category that were assigned to KEGG pathways involved in axon repulsion and attraction, MAPK signaling and Wnt signaling (Figure 3A). Similar organization of protein coding downregulated unigenes was also seen with regard to unigenes assigned to the Regulation of Actin Cytoskeleton (Figure 3B) and Adherens Junction (Figure 3C) GO terms. Transcripts coding for almost the entire portion of the cAMP signaling pathway leading to calcium handling, cytoskeletal rearrangement and gene expression were downregulated (Figure 4A). Many genes that were part of the IP3 pathway for cAMP signaling and calcium handling within the cell were present in the downregulated unigene set, and were related to Calcium signaling (Figure 4C). Genes in key signaling pathways were consistently downregulated during early virus infection, including a significant number of genes comprising the Focal Adhesion pathway, that are part of PI3K signaling (Figure 4B), and other signaling pathways such as Hippo Signaling in Fly (Figure 4D) and Notch Signaling (Figure 4E).

### 3.6. Analysis of Unigenes Associated with Sensory Processes, Behavior, Learning and Memory

**Figure 5:**
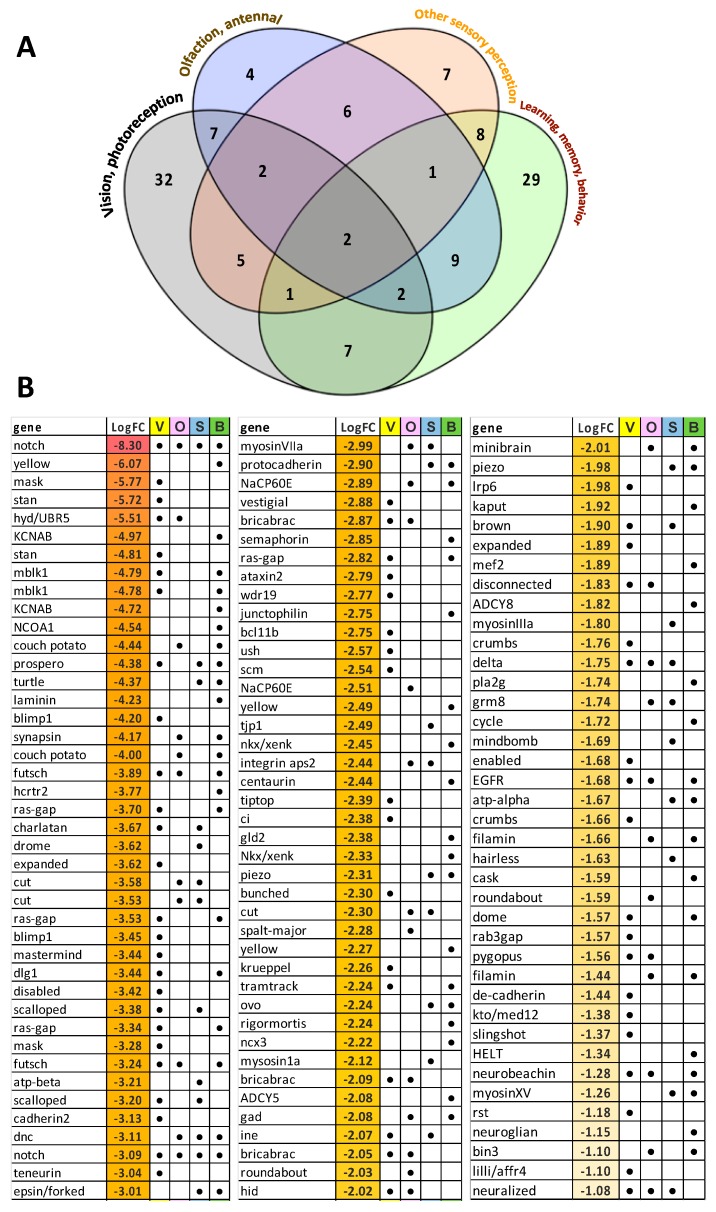
Unigenes associated with sensory processes, behavior, learning and memory that were significantly downregulated in EHDV-2 infected midges. Differentially-expressed unigenes were manually inspected, catalogued and categorized to present sets associated with sensory processes (vision, olfaction, other) and brain functions (learning, memory, behaviors). (**A**) 122 unigenes were downregulated with EHDV-2 infection: 62 associated with vision, 35 associated with olfaction, 32 other sensory functions (e.g., gustatory, touch), 59 brain functions. (**B**) Log_2_FC in expression of unigenes associated with vision (V), olfaction (O), other sensory (S) and brain functions (B) are given. As both panels indicate, many of these unigenes have overlapping functions. Further details including functional roles for each unigene can be found in Supplementary Table S8.

**Figure 6:**
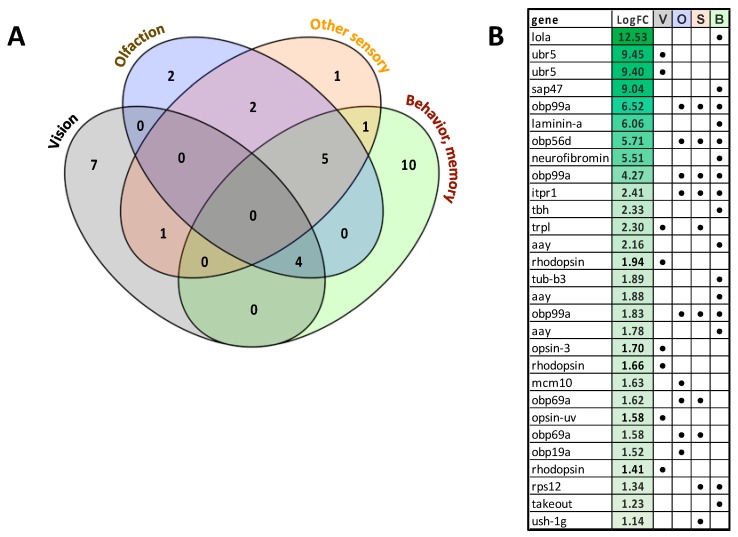
Unigenes associated with sensory processes, behavior, learning and memory that were significantly upregulated in EHDV-2 infected midges. Differentially-expressed unigenes were manually inspected, catalogued and categorized to present sets associated with sensory processes (vision, olfaction, other) and brain functions (learning, memory, behaviors). (**A**) Of the 29 unigenes that were upregulated with EHDV-2 infection: 8 associated with vision, 9 associated with olfaction, 10 associated with other sensory functions (e.g., gustatory, touch), and 16 to brain, memory and/or learning. (**B**) Log_2_-FC in expression of unigenes associated with vision (V), olfaction (O), other sensory (S) and brain functions (B) are given. As both panels indicate, many of these unigenes have overlapping functions. Further details including functional roles for each unigene can be found in Supplementary Table S8.

Manual cataloguing and categorizing of unigenes that were associated with sensory processes, behavior and learning/memory revealed 122 unigenes that were downregulated with EHDV-2 infection including 62 associated with vision, 35 associated with olfaction, 32 linked to other sensory functions (e.g., gustatory, touch) and 59 related to brain function, behavior, memory and/or learning. Many of these unigenes overlapped in categories, as evident in Figure 5. Of the downregulated vision-associated unigenes, a large number were associated with ommatidial structure and photoreceptor morphogenesis and development. Interestingly, some of the downregulated unigenes were involved with functions and processes such as circadian rhythm, sound perception, locomotory response, mechanoperception and nociception. Of note, 18 of the downregulated unigenes were associated with memory. Only 29 unigenes assigned to these four broad categories were upregulated with EHDV-2 infection, including 8 associated with vision, 9 associated with olfaction, 10 linked to other sensory functions and 16 to brain, behavior, memory and/or learning. Again, many of these unigenes had overlapping functions across the four categories (Figure 6). Interestingly, 5 of 8 upregulated vision- associated unigenes coded for the pigments opsin or rhodopsin and 7 of 9 upregulated olfaction-associated unigenes encoded odorant-binding proteins. Further details including functional roles for each unigene can be found in Supplementary Table S8.

## 4. Discussion

A broad goal of this study was to characterize the transcriptional response to early (36 h post-ingestion) EHDV-2 infection in female *C. sonorensis*. From our previous work with this same EHDV serotype [9], as well as other work with the related orbivirus BTV [33], we predicted that the major biological responses in the midge would be related to virus proliferation in the midgut at this early time point after virus ingestion and that many genes would be associated with insect anti-viral defenses. Indeed, we did observe upregulation of unigenes coding for innate immune defense effectors including 4 AMPs and 3 prophenoloxidases. However, the majority of the observed changes in unigene expression pointed towards dissemination of the virus to extra-alimentary tissues such as the nervous system and sensory organs.

Using GO enrichment and subsequent KEGG pathway mapping, we elucidated that midges that had fed on EHDV-2 substantially downregulated unigenes involved in cytoskeletal remodeling, in particular actin remodeling, cAMP pathways and calcium signaling, tissue regeneration (e.g., hippo signaling) and cell proliferation (e.g., notch signaling). Whether these responses were caused by the virus altering gene expression or, alternatively, were a result of viral disruption of tissue function (whereby decreased gene expression in those cells is a side effect of pervasive shutdown of cellular processes triggered by infection) remains unclear. Nonetheless, the resulting expression phenotype is the same irrespective of the underlying, causal cellular mechanism. KEGG analysis also revealed widespread downregulation of unigenes associated with focal adhesion, which would affect overall tissue architecture. Taken together, the transcriptional changes observed 36 h after virus ingestion were consistent with changes to cellular structure (e.g., cytoskeletal changes) and tissue architecture (e.g., effects on cell adhesion and cell-cell junctions), as well as regeneration of damaged tissues. It is intriguing to speculate how these changes in cellular responses might either promote, or prevent, cell-to-cell dissemination and propagation of the virus throughout the midge’s body. This avenue of research deserves further exploration in order to fully understand the cellular mechanisms underlying disseminated EHDV-2 infection. Pairing gene expression analysis with histopathology, especially at an ultrastructural level, could provide valuable information by filling knowledge gaps associated with virus dissemination in the midge.

GO enrichment and subsequent KEGG mapping also revealed significant downregulation of unigenes associated with axon guidance, axon and nerve branching, and axon regeneration in midges that ingested EHDV-2. In our previous study, which tracked EHDV-2 spatial dissemination in the midge over the time course of infection [9], we did not detect EHDV-2 in the midge’s neural network. However, the immunostaining technique used, while specific for the virus, may not have been sensitive enough to detect infection of these tissues, especially at early infection times when virus counts can be very low. Notably, other insect-transmitted viruses may utilize axonal transport and the cell cytoskeleton to disseminate [34,35]. However, because several of the unigenes associated with axonal processes have functional overlap with other central neurological functions such as learning and memory (discussed below), the global downregulation of these genes may not be indicative of alteration of corporal axons but rather those associated with the sensory system’s nerve cell bodies and/or ganglia.

Manual inspection of differentially-expressed unigenes associated with sensory and memory/behavior processes showed an overall downregulation of unigenes related to both the function and structure of key sensory organs and cerebral ganglia. Previously, we detected EHDV-2 in the eye as early as 5 d after ingestion by immunostaining [9]. The current study suggests that the virus could be affecting these tissues as soon as 36 h post-ingestion, as indicated by the downregulation of 62 unigenes associated with vision (majority being related to eye, ommatidial and photoreceptor structure) in midges that ingested EHDV-2 compared to controls. Interestingly, 5 unigenes associated with photosensory pigments (rhodopsin, opsin) were upregulated, likely as a feedback response to loss of function that resulted from the massive downregulation of vision-associated unigenes. We speculate that photoreceptor and other vision-related tissues are not in fact downregulating gene expression but rather that tissue integrity is being altered or disrupted and that this process results in the downregulation of these transcripts. Irrespective of the underlying mechanism, downregulation of genes for key structures and functions in the eye potentially affects visual perception in infected midges.

Interestingly, McDermott et al. [36] showed that BTV infected the ommatidial tissues of *C. sonorensis* and that this tissue tropism likely impacts their attraction to light traps, rendering them light-averse. In our study, not only were visual perception-related unigenes downregulated in infected midges, but several unigenes associated with short- and long-term memory, the startle and pain responses, and circadian rhythm also were downregulated. These changes in gene expression could collectively contribute to profound effects on the behavior of infected midges. Of note, we demonstrated that 7 unigenes coding for odorant-binding proteins, which are often associated with detection of resources, including hosts, were upregulated in EHDV-2 infected midges. Taken together, these findings imply that infection with orbiviruses such as EHDV or BTV may alter the host-seeking and feeding behavior of the midge. Such phenotypic effects could only be demonstrated by well-designed behavioral studies which, to our knowledge, have yet to be performed. Nonetheless, pathogen modification of host-seeking or feeding behaviors in infected vectors is not a novel concept and has been observed in a wide variety of other vector-pathogen systems [37,38,39,40,41].

Our study was the first to use a next-gene sequencing approach (RNAseq) to elucidate the genetic response to EHDV infection in the midge. In comparison, a previous study by Campbell and Wilson [42] used differential display RT-PCR and subtractive hybridization techniques to identify genes upregulated in the *C. sonorensis* midgut in response to ingestion of another EHDV serotype (EHDV-1), also focusing at early time points after infection (1 and 2 days). Remarkably, we did not see any common differentially-expressed transcripts between the two studies, and none of the genes upregulated in the previous study were upregulated in midges fed EHDV-2 in our study. The lack of similarity between the previous and current study may be attributable to differences in virus serotype (EHDV-1 vs. EHDV-2), *C. sonorensis* colony strains, the method for feeding (fetal bovine serum vs. sheep blood) and the tissue of focus (midgut vs. whole midges). The discrepancies between these two studies illuminate the need for additional investigations into midge responses to other strains of EHDV as well as other orbiviruses. Incorporation of wild-caught midges in study design and feeding midges on viremic animals rather than artificial feeding systems will help in approximating the real effects that orbivirus infections impart on *Culicoides* spp.

## 5. Conclusions

We described a transcriptome-wide response to EHDV-2 infection in the midge. Surprisingly, response to early infection did not elucidate any obvious barriers to infection that may underlie vector competence, apart from the upregulation of a handful of unigenes coding for innate immune components. Instead, our analyses revealed system-wide effects on gene expression which indicated that dissemination may occur as soon as 36 h post-ingestion. We saw a bias towards downregulation of gene expression, with many unigenes being ascribed to pathways associated with cell and tissue structure and integrity, axon growth and function, sensory processes (especially vision) and brain functions, such as memory and behavior. The possibility of behavioral modification of EHDV-2 infected midges should be explored in future studies in order to better understand how infection impacts host seeking and feeding in this important vector, which could help improve design of collection and control methods.

## Figures and Tables

**Table 1:** Virus isolation from EHDV-infected midges.

Time (h)	% CPE Positive *	TCID_50_ per midge (*n*) **
0	100 (10/10)	2.97 (4), 3.1 (3), 3.3 (3)
36	100 (10/10)	<2.3 (6), 2.3 (2), 2.5, 2.8

* no. positive/*n* ** Log_10_/midge, *n* is denoted if *n* > 1.

## Supplementary Materials

The following are available online at https://www.mdpi.com/1999-4915/11/5/473/s1, Table S1. Primer sequences used for qRT-PCR in female *C. sonorensis;* Table S2. *Culicoides sonorensis* reference gene and amino acid sequences; Table S3. *Culicoides sonorensis* upregulated unigenes 36 h post-ingestion of EHDV-2, relative to control blood meal; Table S4. *Culicoides sonorensis* downregulated unigenes 36 h post-ingestion of EHDV-2, relative to control blood meal; Table S5. GO Enrichment Upregulated; Table S6. GO Enrichment Downregulated; Table S7A and S7B. Biologically relevant GO terms upon EHDV-2 ingestion; Table S8A. Downregulated unigenes associated with sensory processes, behavior, learning and memory; Table S8B. Upregulated unigenes associated with sensory processes, behavior, learning and memory; Figure S1: qRT-PCR validation of selected differentially-expressed unigenes.

## References

[1] Ruder M.G., Lysyk T.J., Stallknecht D.E., Foil L.D., Johnson D.J., Chase C.C., Dargatz D.A., Gibbs E.P.J. (2015). Transmission and epidemiology of bluetongue and epizootic hemorrhagic disease in North America: Current perspectives, research gaps, and future directions. Vector-Borne Zoonotic Dis..

[2] Ruder M.G., Johnson D., Ostlund E., Allison A.B., Kienzle C., Phillips J.E., Poulson R.L., Stallknecht D.E. (2017). The first 10 years (2006–15) of epizootic hemorrhagic disease virus serotype 6 in the USA. J. Wildl. Dis..

[3] Beringer J., Hansen L.P., Stallknecht D.E. (2000). An epizootic of hemorrhagic disease in white-tailed deer in Missouri. J. Wildl. Dis..

[4] Stevens G., McCluskey B., King A., O’Hearn E., Mayr G. (2015). Review of the 2012 epizootic hemorrhagic disease outbreak in domestic ruminants in the United States. PLoS ONE.

[5] Gaydos J.K., Crum J.M., Davidson W.R., Cross S.S., Owen S.F., Stallknecht D.E. (2004). Epizootiology of an epizootic hemorrhagic disease outbreak in West Virginia. J. Wildl. Dis..

[6] McGregor B.L., Erram D., Acevedo C., Alto B.W., Burkett-Cadena N.D. (2019). Vector competence of *Culicoides sonorensis* (Diptera: Ceratopogonidae) for epizootic hemorrhagic disease virus serotype 2 strains from Canada and Florida. Viruses.

[7] Mendiola S.Y., Mills M.K., Maki E., Drolet B.S., Wilson W.C., Berghaus R., Stallknecht D.E., Breitenbach J., McVey D.S., Ruder M.G. (2019). EHDV-2 infection prevalence varies in *Culicoides sonorensis* after feeding on infected white-tailed deer over the course of viremia. Viruses.

[8] Ruder M.G., Howerth E.W., Stallknecht D.E., Allison A.B., Carter D.L., Drolet B.S., Klement E., Mead D.G. (2012). Vector competence of *Culicoides sonorensis* (Diptera: Ceratopogonidae) to epizootic hemorrhagic disease virus serotype 7. Parasit. Vectors.

[9] Mills M.K., Ruder M.G., Nayduch D., Michel K., Drolet B.S. (2017). Dynamics of epizootic hemorrhagic disease virus infection within the vector, *Culicoides sonorensis* (Diptera: Ceratopogonidae). PLoS ONE.

[10] Mills M.K., Michel K., Pfannenstiel R.S., Ruder M.G., Veronesi E., Nayduch D. (2017). *Culicoides*–virus interactions: Infection barriers and possible factors underlying vector competence. Curr. Opin. Insect Sci..

[11] Nayduch D., Lee M.B., Saski C.A. (2014). The reference transcriptome of the adult female biting midge (*Culicoides sonorensis*) and differential gene expression profiling during teneral, blood, and sucrose feeding conditions. PLoS ONE.

[12] Nayduch D., Lee M.B., Saski C.A. (2014). Gene discovery and differential expression analysis of humoral immune response elements in female *Culicoides sonorensis* (Diptera: Ceratopogonidae). Parasit Vectors.

[13] Cheng G., Liu Y., Wang P., Xiao X. (2016). Mosquito defense strategies against viral infection. Trends Parasitol..

[14] Zhao P., Lu Z., Strand M.R., Jiang H. (2011). Antiviral, anti-parasitic, and cytotoxic effects of 5, 6-dihydroxyindole (DHI), a reactive compound generated by phenoloxidase during insect immune response. Insect Biochm. Mol. Biol..

[15] Rodriguez-Andres J., Rani S., Varjak M., Chase-Topping M.E., Beck M.H., Ferguson M.C., Schnettler E., Fragkoudis R., Barry G., Merits A. (2012). Phenoloxidase activity acts as a mosquito innate immune response against infection with Semliki Forest virus. Plos Pathog..

[16] Kingsolver M.B., Huang Z., Hardy R.W. (2013). Insect antiviral innate immunity: Pathways, effectors, and connections. J. Mol. Biol..

[17] Nunamaker R.A., De León A.A.P., Campbell C.L., Lonning S.M. (2000). Oral infection of *Culicoides sonorensis* (Diptera: Ceratopogonidae) by vesicular stomatitis virus. J. Med. Entomol..

[18] Reed L.J., Muench H. (1938). A simple method of estimating fifty per cent endpoints. Am. J. Epidemiol..

[19] Bolger A.M., Lohse M., Usadel B. (2014). Trimmomatic: A flexible trimmer for Illumina sequence data. Bioinformatics.

[20] Grabherr M.G., Haas B.J., Yassour M., Levin J.Z., Thompson D.A., Amit I., Adiconis X., Fan L., Raychowdhury R., Zeng Q. (2011). Full-length transcriptome assembly from RNA-Seq data without a reference genome. Nat. Biotechnol..

[21] Li W., Godzik A. (2006). Cd-hit: A fast program for clustering and comparing large sets of protein or nucleotide sequences. Bioinformatics.

[22] El-Gebali S., Mistry J., Bateman A., Eddy S.R., Luciani A., Potter S.C., Qureshi M., Richardson L.J., Salazar G.A., Smart A. (2018). The Pfam protein families database in 2019. Nucleic Acids Res..

[23] Consortium T.U. (2018). UniProt: A worldwide hub of protein knowledge. Nucleic Acids Res..

[24] Wu T.D., Watanabe C.K. (2005). GMAP: A genomic mapping and alignment program for mRNA and EST sequences. Bioinformatics.

[25] Liao Y., Smyth G.K., Shi W. (2014). featureCounts: An efficient general purpose program for assigning sequence reads to genomic features. Bioinformatics.

[26] Robinson M.D., McCarthy D.J., Smyth G.K. (2010). edgeR: A Bioconductor package for differential expression analysis of digital gene expression data. Bioinformatics.

[27] Benjamini Y., Hochberg Y. (1995). Controlling the false discovery rate: A practical and powerful approach to multiple testing. J. R. Statist. Soc..

[28] Young M.D., Wakefield M.J., Smyth G.K., Oshlack A. (2010). Gene ontology analysis for RNA-seq: Accounting for selection bias. Genome Biol..

[29] Pfaffl M.W., Horgan G.W., Dempfle L. (2002). Relative expression software tool (REST©) for group-wise comparison and statistical analysis of relative expression results in real-time PCR. Nucleic Acids Res..

[30] Volders K., Scholz S., Slabbaert J.R., Nagel A.C., Verstreken P., Creemers J.W., Callaerts P., Schwärzel M. (2012). *Drosophila* rugose is a functional homolog of mammalian Neurobeachin and affects synaptic architecture, brain morphology, and associative learning. J. Neurosci..

[31] Giniger E., Tietje K., Jan L.Y., Jan Y.N. (1994). *lola* encodes a putative transcription factor required for axon growth and guidance in *Drosophila*. Development.

[32] Prokop A., Beaven R., Qu Y., Sánchez-Soriano N. (2013). Using fly genetics to dissect the cytoskeletal machinery of neurons during axonal growth and maintenance. J. Cell Sci..

[33] Fu H., Leake C.J., Mertens P.P., Mellor P.S. (1999). The barriers to bluetongue virus infection, dissemination and transmission in the vector, *Culicoides variipennis* (Diptera: Ceratopogonidae). Arch. Virol..

[34] Drolet B.S., Campbell C.L., Stuart M.A., Wilson W.C. (2005). Vector competence of *Culicoides sonorensis* (Diptera: Ceratopogonidae) for vesicular stomatitis virus. J. Med. Entomol..

[35] Ammar E.-D., Hogenhout S.A. (2008). A neurotropic route for Maize mosaic virus (Rhabdoviridae) in its planthopper vector *Peregrinus maidis*. Virus Res..

[36] McDermott E.G., Mayo C.E., Gerry A.C., Laudier D., MacLachlan N.J., Mullens B.A. (2015). Bluetongue virus infection creates light averse *Culicoides* vectors and serious errors in transmission risk estimates. Parasit. Vectors.

[37] Grimstad P.R., Ross Q.E., Craig G.B. (1980). *Aedes triseriatus* (Diptera: Culicidae) and La Crosse Virus: II. Modification of mosquito feeding behavior by virus infection. J. Med. Entomol..

[38] Platt K.B., Linthicum K.J., Myint K.S., Innis B.L., Lerdthusnee K., Vaughn D.W. (1997). Impact of dengue virus infection on feeding behavior of *Aedes aegypti*. Am. J. Trop. Med. Hyg..

[39] Lefcort H., Durden L.A. (1996). The effect of infection with Lyme disease spirochetes (Borrelia burgdorferi) on the phototaxis, activity, and questing height of the tick vector *Ixodes scapularis*. Parasitology.

[40] Beach R., Kiilu G., Leeuwenburg J. (1985). Modification of sand fly biting behavior by *Leishmania* leads to increased parasite transmission. Am. J. Trop. Med. Hyg..

[41] Jackson B.T., Brewster C.C., Paulson S.L. (2014). La Crosse virus infection alters blood feeding behavior in *Aedes triseriatus* and *Aedes albopictus* (Diptera: Culicidae). J. Med. Entomol..

[42] Campbell C.L., Wilson W.C. (2002). Differentially expressed midgut transcripts in *Culicoides sonorensis* (Diptera: Ceratopogonidae) following Orbivirus (Reoviridae) oral feeding. Insect Mol. Biol..

