# Supplementary material for: Transcriptome Response of Female Culicoides sonorensis Biting Midges (Diptera: Ceratopogonidae) to Early Infection with Epizootic Hemorrhagic Disease Virus (EHDV-2)
Source: Viruses. 2019 May 24;11(5):473. doi: 10.3390/v11050473 (PMC6563219; doi:10.3390/v11050473)
Supplement: Supplementary file 1 [file viruses-11-00473-s001.zip › viruses-505912-supplementary-english/Figure S1.pdf]

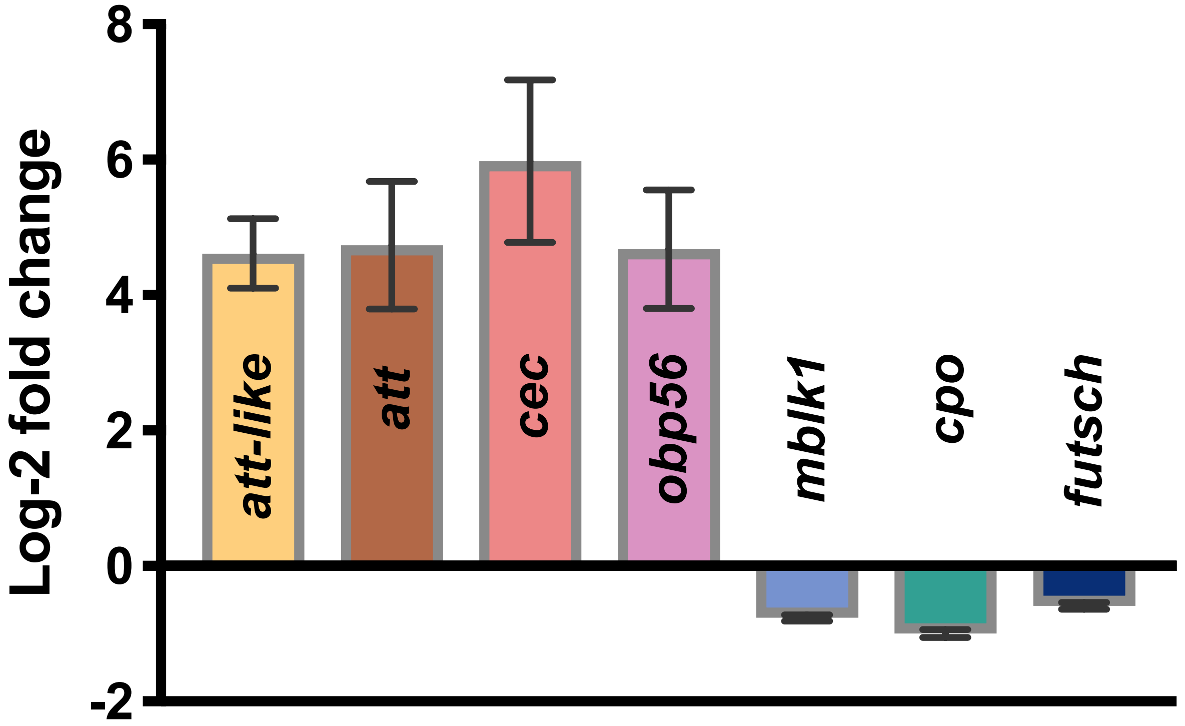

Figure S1. qRTPCR validation of selected differentially-expressed unigenes.
Changes in gene expression between EHDV-2 fed or control blood meal fed midges (n=15). Primer sequences are in Table S1 and qRTPCR details are in the text. Fold change in expression calculated using the Relative Expression Software tool, allowing for group wise comparison and statistical analysis of relative expression while accounting for different primer efficiencies and calculating associated pairwise fixed reallocation randomization with error (bars shown; see reference [23]). *att-like* = attacin-like antimicrobial peptide; *att* = attacin; *cec* = cecropin; *obp56* = odorant-binding protein 56; *mblk1* = mushroom body large-type Kenyon cell-specific protein 1; *cpo* = couch potato; *futsch* = microtubule associated protein futsch.
